# Targeting the A_3_ adenosine receptor to prevent and reverse chemotherapy-induced neurotoxicities in mice

**DOI:** 10.1186/s40478-022-01315-w

**Published:** 2022-01-29

**Authors:** Anand Kumar Singh, Rajasekaran Mahalingam, Silvia Squillace, Kenneth A. Jacobson, Dilip K. Tosh, Shruti Dharmaraj, Susan A. Farr, Annemieke Kavelaars, Daniela Salvemini, Cobi J. Heijnen

**Affiliations:** 1 Department of Symptom Research, The University of Texas MD Anderson Cancer Center, Houston, TX USA; 2 Department of Pharmacology and Physiology, Saint Louis University School of Medicine, St. Louis, MO USA; 3 The Henry and Amelia Nasrallah Center for Neuroscience, Saint Louis University School of Medicine, St. Louis, MO USA; 4 Department of Internal Medicine-Geriatrics, Saint Louis School of Medicine, St. Louis, MO USA; 5 Research & Development Service, VA Medical Center, St. Louis, MO USA; 6 National Institute of Diabetes and Digestive and Kidney Disease, National Institutes of Health, Bethesda, MD 20892 USA

**Keywords:** Chemotherapy, Cisplatin, Adenosine, A3AR, Cognitive impairment, Sensorimotor deficit, Peripheral neuropathy, Mechanical allodynia, Spontaneous pain

## Abstract

Cisplatin is used to combat solid tumors. However, patients treated with cisplatin often develop cognitive impairments, sensorimotor deficits, and peripheral neuropathy. There is no FDA-approved treatment for these neurotoxicities. We investigated the capacity of a highly selective A_3_ adenosine receptor (AR) subtype (A_3_AR) agonist, MRS5980, to prevent and reverse cisplatin-induced neurotoxicities. MRS5980 prevented cisplatin-induced cognitive impairment (decreased executive function and impaired spatial and working memory), sensorimotor deficits, and neuropathic pain (mechanical allodynia and spontaneous pain) in both sexes. At the structural level, MRS5980 prevented the cisplatin-induced reduction in markers of synaptic integrity. In-situ hybridization detected *Adora3* mRNA in neurons, microglia, astrocytes and oligodendrocytes. RNAseq analysis identified 164 genes, including genes related to mitochondrial function, of which expression was changed by cisplatin and normalized by MRS5980. Consistently, MRS5980 prevented cisplatin-induced mitochondrial dysfunction and decreased signs of oxidative stress. Transcriptomic analysis showed that the A_3_AR agonist upregulates genes related to repair pathways including NOTCH1 signaling and chromatin modification in the cortex of cisplatin-treated mice. Importantly, A_3_AR agonist administration after completion of cisplatin treatment resolved cognitive impairment, neuropathy and sensorimotor deficits. Our results highlight the efficacy of a selective A_3_AR agonist to prevent and reverse cisplatin-induced neurotoxicities via preventing brain mitochondrial damage and activating repair pathways. An A_3_AR agonist is already in cancer, clinical trials and our results demonstrate management of neurotoxic side effects of chemotherapy as an additional therapeutic benefit.

**Supplementary Information:**

The online version contains supplementary material available at 10.1186/s40478-022-01315-w.

## Introduction

Up to seventy-five percent of cancer survivors treated with chemotherapy suffer from neurotoxic side effects, including cognitive deficits, reduced motor function and peripheral neuropathy that can persists for years after completion of treatment [19, 22]. Due to improved treatment options, the number of cancer survivors suffering from neurotoxicities is increasing, but no efficacious FDA-approved interventions addressing this unmet need exist [22, 42, 47].

In search for novel treatment options, we focused on agonists of the A_3_ adenosine receptor (AR) subtype (A_3_AR) for two reasons. First, preclinical studies have shown that A3AR agonists can prevent and reverse cognitive deficits induced by traumatic injury to the brain [14] and peripheral and neuropathic pain after nerve injury [5]. These neuroprotective effects are thought to be mediated through suppression of neuroinflammation and induction of IL10 production [23]. Second, A_3_AR are overexpressed in tumor cells and A_3_AR agonists inhibit the growth of a wide range of cancer cells in vivo and in vitro [15, 37]. The mechanism of anti-tumor effects involves inhibition of telomerase activity and cell cycle arrest in the G0/G1 phase, down-regulation of nuclear factor kappa-B and cyclin D1 and enhanced NK cell-mediated cytotoxicity [18, 37, 39]. A_3_AR agonists already demonstrating good safety profiles in the clinical trials and the US Food and Drug Administration (FDA) and the European Medicines Agency have granted fast track designation to an A_3_AR agonist for the treatment of hepatocellular carcinoma [21].

We recently developed a mouse model of cisplatin-induced cognitive impairments and peripheral neuropathic pain. Treatment of mice with a cumulative dose of 23 mg/kg of cisplatin (human equivalent dose 70 mg/m2) induces deficits in executive functioning and in short term and spatial memory [7, 8, 40]. These cognitive deficits are associated with a loss of synaptic proteins in the CA1 and dentate gyrus of the hippocampus, and a reduction in dendritic spine density in the cingulate cortex that are induced by synaptosomal mitochondrial dysfunction [9, 34, 57]. This cisplatin regimen also induces peripheral neuropathy characterized by mechanical allodynia and spontaneous pain [26, 38]. These signs of peripheral neuropathy are associated with a loss of intra-epidermal nerve (IENF) fibers, changes in mitochondrial morphology in the dorsal root ganglia (DRG) as analyzed by transmission electron microscope (TEM), and decreases in mitochondrial membrane potential and reduced oxygen consumption rate in DRG and peripheral nerves [35, 36, 38].

Using our model of cisplatin-induced cognitive deficits and peripheral neuropathy we tested the capacity of the novel, highly selective A_3_AR agonist MRS5980 to prevent and/or reverse cisplatin-induced cognitive impairment, sensorimotor deficits, the associated reduction in synaptic integrity and mitochondrial abnormalities in the brain. We also examined the effect of MRS5980 on mechanical allodynia and spontaneous pain induced by cisplatin. In addition, we used RNAscope in situ hybridization to investigate the cellular localization of brain A_3_AR receptors and performed RNAseq analysis to identify potential pathways involved in the protective effects of MRS5980.

## Materials and methods

### Animals

Male and female C57BL/6 J mice (8–12 weeks) were purchased from Jackson Laboratory and housed in MD Anderson Cancer Center animal facility on a 12/12 h reverse day-light cycle at 22 ± 2 °C with water and food ad libitum. All experimental procedures were consistent with the National Institute of Health Guidelines for the Care and Use of Laboratory Animals and were approved by the Institutional Animal Care and Use Committee of MD Anderson Cancer Center, Houston, TX. Mice were randomly assigned to experimental groups and all behavioral tests were performed by an investigator blinded for treatment.

### Drugs

Mice were treated with cisplatin in phosphate buffered saline (PBS) or vehicle for two cycles of 5 daily doses of 2.3 mg/kg, i.p with five days of rest in between. This treatment regimen has antitumor effects in the mouse and induces cognitive deficits [7, 8]. MRS5980 was synthesized as described previously [49] and dissolved in 2.5% dimethyl sulfoxide (DMSO) in sterile saline and was injected i.p. daily from one day before the start of cisplatin treatment until one day after the last cisplatin dose. Two weeks after treatment completion, mice were tested in behavioral tests for cognitive-, sensorimotor function, and neuropathic pain.

### Puzzle box test (PBT)

The puzzle box test for executive functioning was performed as described previously [2, 7, 8]. Mice were placed individually into a brightly illuminated arena from which they can escape to a dark goal box by a tunnel. Time to reach the goal box was assessed when the tunnel was open (easy trials 1–4), filled with bedding (intermediate trials 5–7) or blocked with a plug (difficult trials 8–11).

### Novel object place recognition test (NOPRT)

The NOPRT for short term memory and place recognition was performed as described [9]. During training, mice were introduced to two identical objects placed on one side of a rectangular arena for five min. After 1 h, mice were returned to the arena which containing one now familiar object at the same location as in the training session, and one novel object (Rubik’s cube) placed in a novel location. Interaction times with each of the objects were recorded during 5 min. and analyzed with EthoVision XT 10.1 video tracking software (Noldus Information Technology Inc., Leesburg, VA). The discrimination index was calculated as (T_Novel_ − T_Familiar_)/(T_Novel_ + T_Familiar_).

### Y-maze

The Y-maze test for spatial memory was performed in a maze consisting of 3 arms (35 cm length × 5 cm width × 15.5 cm height per arm) at 120-degree angles as described [9]. Each mouse was individually placed in one arm and allowed to freely explore the y-maze for 5 min. The percentage of perfect alternations (entry into an arm that differs from the previous two entries) over total arm entries was calculated as the ratio of the number of perfect alternations to the total number of possible alternations.

### Sensorimotor function

In the beam walk test for sensorimotor function mice were trained on 3 consecutive days to cross a wide rectangular beam (3 trials), followed by a narrow rectangular or round beam until control mice successfully crossed the beam. The time to cross was then determined in 2 videotaped trials analyzed by investigators blinded to treatment [7].

### Mechanical allodynia

Mice were placed in plastic cages on a mesh stand (IITC Life Science, Woodland Hills, CA) and 50% withdrawal thresholds were determined using von Frey filaments (0.02, 0.07, 0.16, 0.4, 0.6, 1.0, and 1.4 g) (Stoelting, Wood Dale, Illinois, USA) and the up and down method [4, 28, 36].

### Spontaneous pain

Spontaneous pain was assessed in a conditioned place preference (CPP) test with retigabine as the conditioning stimulus [26, 28]. During preconditioning each mouse was allowed to explore the CPP apparatus consisting of a dark and a light chamber connected by a hallway for 15 min (Stoelting, Wood Dale, IL). During the 4 days of conditioning mice were injected with PBS and confined to the dark chamber for 20 min in the morning and injected with analgesic, retigabine (R-100, Alomone Laboratory, Jerusalem, Israel;10 mg/kg i.p) and confined to the bright chamber for 20 min in the afternoon. On the test day, drug-free mice explored the apparatus for 15 min, and the time spent in the bright (previously analgesic-paired) chamber was quantified. An increase in the time spent in the bright chamber from baseline to test was interpreted as evidence of spontaneous pain.

### RNAscope in situ hybridization

RNAscope in situ hybridization was performed on 10 µm brain section using RNAscope® Multiplex Fluorescent Reagent Kit v2 (Advanced Cell Diagnostics). Briefly, sections were fixed in 10% normal buffered formalin (pH 7.4) at 4 $^\circ$C, dehydrated, treated with hydrogen peroxide for 10 min followed by protease IV for 30 min at RT and incubated with Channel 1 (*Adora3*), Channel 2 (*Map2* or GFAP), and Channel 3 (*Tmem119* or *Olig2*) probes (50:1:1 dilution) for 2 h in a pre-warmed humidity control tray (ACD) at 40 $^\circ$C. After 3 sequential amplifications followed by conjugation of fluorophore for each channel (channel 1: Opal 520; Akoya Biosciences; OP-001001, channel 2: Opal 620; Akoya Biosciences; OP-001004 and channel 3: Opal 680; Akoya Biosciences; OP-001006). Slides were stained with DAPI (200 µl/ slide, ACD), cover slipped in Prolong Gold Antifade mounting medium and air-dried overnight. Images were captured using a confocal microscope (Nikon) at 40 × and 100 × magnification. Images were brightened and contrasted in Adobe Photoshop and *Adora3*-positive neurons, microglia, astrocytes, and oligodendrocytes were counted manually. Eight images of 40X frame per mouse were counted (2 images from each hemisphere, one from primary somatosensory cortex and one from entorhinal cortex on 2 sections per mouse) [43].

### Immunofluorescence analysis

At the end of the behavioral analysis, mice were perfused transcardially with ice-cold PBS with 5 U/mL sodium heparin (Hospira). Brains were post-fixed in 4% PFA for 48 h, cryo-protected in sucrose, and 8 µm coronal sections mounted on SuperFrost slides (Fisher Scientific; 12–550-15), were post-fixed in 4% PFA for 1 h, blocked in 2% bovine serum albumin, 10% normal goat serum and 0.1% saponin in PBS and incubated with rabbit anti-synaptophysin (1:1000, Millipore; AB9272) or rabbit anti-PSD95 (1:1000, Abcam; AB18258), followed by Alexa 488 goat anti-rabbit (1:500, Invitrogen; A-11043) or Alexa 647 goat anti rabbit (1:500, Thermo Fisher Scientific; A-21245). As a negative control, the primary antibody was omitted. Fluorescence was visualized using the Nikon A1R Confocal Microscope (Nikon Instruments Inc., Melville, NY, USA) using 40X objective. The average number of synaptophysin and PSD95 positive puncta were quantified in the CA1 of the hippocampus in three regions of interest using the spot detection feature of the Nikon NIS-Elements Advanced Research Software (Nikon Instruments Inc., Melville, NY, USA).

### Mitochondrial bioenergetics

Synaptosomal mitochondrial bioenergetics were analyzed using Seahorse technology [9]. Synaptosomes prepared from one hemisphere using the Syn-PER extraction reagent (Thermo Scientific, 87793) with Halt protease and phosphatase inhibitor cocktail (Thermo Scientific, 78440) were resuspended in base media (Agilent Technologies, Santa Clara, CA) supplemented with 11 mM glucose, 2 mM glutamine, and 1 mM pyruvate and plated in a Seahorse XFe 24 microplate (Agilent Technologies, Santa Clara, CA) pre-coated with GelTrex (Life Technologies/ Thermo Fisher Scientific, Waltham, MA). Oxygen consumption rate at baseline and after addition of 4 μM oligomycin (ATP production related oxygen consumption), 1 μM carbonyl cyanide 4-(trifluoromethoxy) phenylhydrazone (FCCP) (maximal respiratory capacity), and 2 μM of rotenone and 2 μM of antimycin A (non-mitochondrial oxygen consumption) were determined. All values were corrected for non-mitochondrial oxygen consumption.

### Immunoprecipitation and western blot analyses

To quantify nitrosylated MnSOD, nitrotyrosine-containing proteins were immunoprecipitated from 500 µg of cortex protein in immunoprecipitation buffer (20 mM Tris-base, 150 mM NaCl, 10% glycerol, 0.1% Triton X-100, 2 mM EGTA, 1% protease inhibitor cocktail) with 10 µg of protein G sepharose beads, 2 µg anti-nitrotyrosine monoclonal antibodies (Millipore, Cat No. 05-233) washed in PBS (pH 7.4). Immunoprecipitates were resuspended in 40 µL of sample buffer (2X, 0.5 M Tris–HCl (pH 6.8), 2.5% glycerol, 0.5% SDS, 200 mM 2-mercaptoethanol, 0.001% bromophenol blue) and resolved in 12% SDS-PAGE separated by SDS-PAGE and transferred to PVDF membranes. The membranes were blocked 3% bovine serum albumin (BSA), 0.01% Tween-20 (TBS/T) in Tris buffered saline, followed by incubation with rabbit-anti-MnSOD (1:1000; Millipore Cat No. 06–984). Membranes followed by horseradish peroxidase coupled secondary antibody (1:5000; Cell Signaling #7074) and visualized by enhanced chemiluminescence (ImageQuant LAS 4000, GE Healthcare). Total lysate was run in parallel and β-actin (1:5000; Sigma, Cat No. A5441) in these samples was for normalization. Protein bands were quantified by densitometry using ImageJ software.

### RNAseq

RNA was extracted from cortex tissues from 3 female mice per sample, 4 samples per group (control, cisplatin, cisplatin + MRS5980 and MRS5980). rRNA was removed using the Ribo-Zero kit to obtain cleaned mRNA. The cleaned mRNA is fragmented randomly by adding fragmentation buffer, and cDNA was synthesized by using mRNA template and random hexamer primers.

The raw reads in FASTQ format were used for the analysis. The quality of the raw reads was analyzed using the FastQC tool. The mouse genome (mm10) was used as a reference for mapping the reads using the STAR package [12]. Gene counts were estimated from uniquely mapped reads using the FeatureCounts program from the Subread tool [30]. We used the DEseq2 program [33] to normalize the gene counts. Genes with < 10 reads were removed and differentially expressed genes were identified with p < 0.05 as the cut off. The GO biological process enrichment analysis was performed using the up-regulated genes from cisplatin + MRS5980 vs cisplatin comparison with the Enrichr program [56].

### Statistical analysis

Data were analyzed using GraphPad Prism version 8.0.0 for Windows (GraphPad Software, San Diego, CA, USA). Error bars indicate SEM and statistical significance was assessed by t-test or by one-way or two-way ANOVA followed by two-tailed Tukey's test for post hoc pair-wise, multiple-comparisons. P < 0.05 was considered statistically significant.

## Results

### Prevention of cisplatin-induced cognitive deficits by the A_3_AR agonist MRS5980

**Fig. 1:**
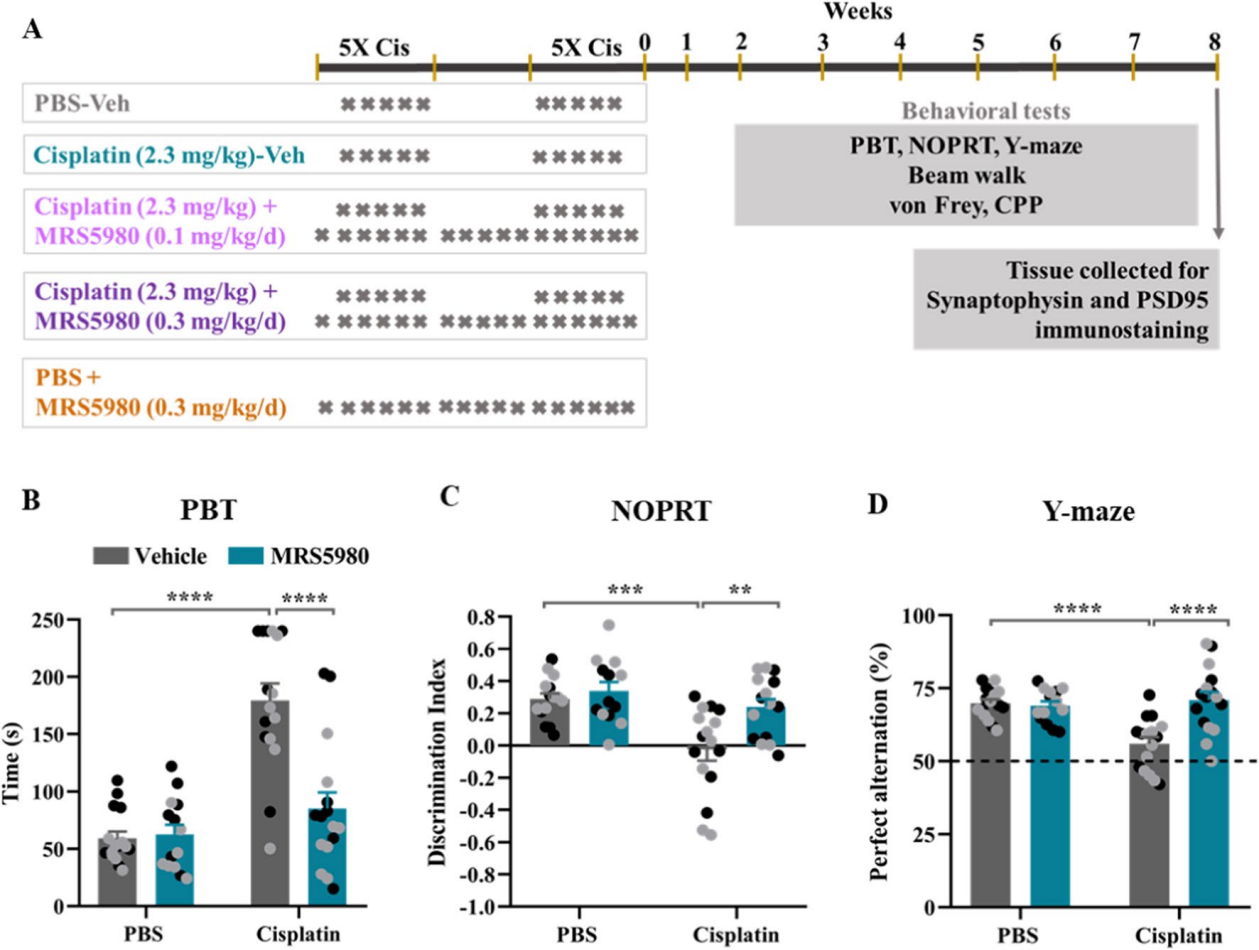
Prevention of cisplatin-induced cognitive deficits by the A3AR agonist MRS5980. **a** Male and female mice were treated with PBS or cisplatin (2 rounds of 5 daily doses of 2.3 mg/kg i.p. with 5 days of rest in between). MRS5980 was given daily (0.3 mg/kg/day i.p.), starting one day before cisplatin administration until one day after the last cisplatin injection. Behavioral tests were started 2 weeks after the last dose of cisplatin in the order listed and tissue was collected for immunostaining after completion of all tests. **b** Influence of MRS5980 on cisplatin-induced deficits in executive function as assessed using the puzzle box test (PBT). Data are presented as mean of time (s) used in the hard trials in which mice have to remove a plug blocking the tunnel to the dark compartment in order to escape from the light compartment. **c** Effect of cisplatin and MRS5980 on working memory in the novel object place recognition test (NOPRT). Preference for the novel object in the NOPRT is presented as discrimination index (DI: (Tnovel—Tfamiliar)/ (Tnovel + Tfamiliar). **d** Influence of cisplatin and MRS5980 on spatial memory in the Y-maze expressed as a percentage of perfect alternations in the Y-maze. Male (black circle; n = 7–8/group) and female (grey circle; n = 6–8/group) mice. Results are expressed as mean ± SEM; Two-way ANOVA with Tukey's post-hoc analysis **p ≤ 0.01; ***p ≤ 0.001; ****p ≤ 0.0001. No significant sex effects were detected

We tested the highly selective A_3_AR agonist MRS5980 [21] for its capacity to prevent cognitive dysfunction induced by two cycles of cisplatin (5 daily doses of 2.3 mg/kg, i.p with five days of rest in between (Fig. 1A). MRS5980 was injected daily from one day before the start of cisplatin until one day after the last dose of cisplatin (Fig. 1A). Executive function was analyzed in the puzzle box test (PBT) two weeks after completion of cisplatin/ MRS5980 treatment. In this test, mice can escape a brightly lit arena to a dark goal box via a tunnel that is open (easy trials), filled with bedding (intermediate trials) or covered with a plug (hard trials). In line with our previous studies [7, 8], cisplatin treatment significantly increased the time needed to reach the goal box during the hard trial indicating impaired executive function (Fig. 1B). In males and females co-administration of MRS5980 at a dose of 0.3 mg/kg/day prevented this adverse effect of cisplatin, while 0.1 mg/kg of MRS5980 was only partially effective (Fig. 1B, Additional File 3: Supplementary figure S1). Consistent with our earlier studies, cisplatin did not affect the time taken to reach the goal box during easy and intermediate trials, indicating mice were equally motivated to enter the dark chamber (Additional File 3: Supplementary figure S2 A-B). MRS5980 alone did not affect performance of male and female mice in the PBT (Fig. 1B, Additional File 3: Supplementary figure S2 A-B).

In the NOPRT, which is a test for spatial and working memory, mice are exposed to two identical objects during the training phase. One hour later, mice are exposed to one of the now familiar objects in the same location and a novel object in a different location. Healthy mice will spend more time with the new object in the new location due to their preference for novelty [9]. Cisplatin treatment reduced the preference for the novel object, indicating impaired working and spatial memory (Fig. 1C). Co-administration of MRS5980 treatment prevented the cisplatin-induced deficits in working and spatial memory in male and female mice (Fig. 1C). There was no effect of cisplatin or MRS5980 on total interacting time with the objects, and MRS5980 alone did not affect the preference for the novel object (Additional File 3: Supplementary figure S2C). Next, we tested spatial memory of mice in the Y-maze test. This test measures the willingness of mice to explore new environments leading to spontaneous alternation when entering the three arms of the Y-maze. Cisplatin reduced the percentage of perfect alternations in male and female mice (Fig. 1D) indicating impaired spatial memory. MRS5980 prevented the cisplatin-induced reduction in spatial memory in both sexes (Fig. 1D). There were no significant differences in the number of total entries in the arms of the Y-maze (Additional File 3: Supplementary figure S2D), indicating that the motivation of the mice to perform the test was similar in all groups.

### MRS5980 prevents cisplatin-induced sensorimotor deficits

**Fig. 2:**
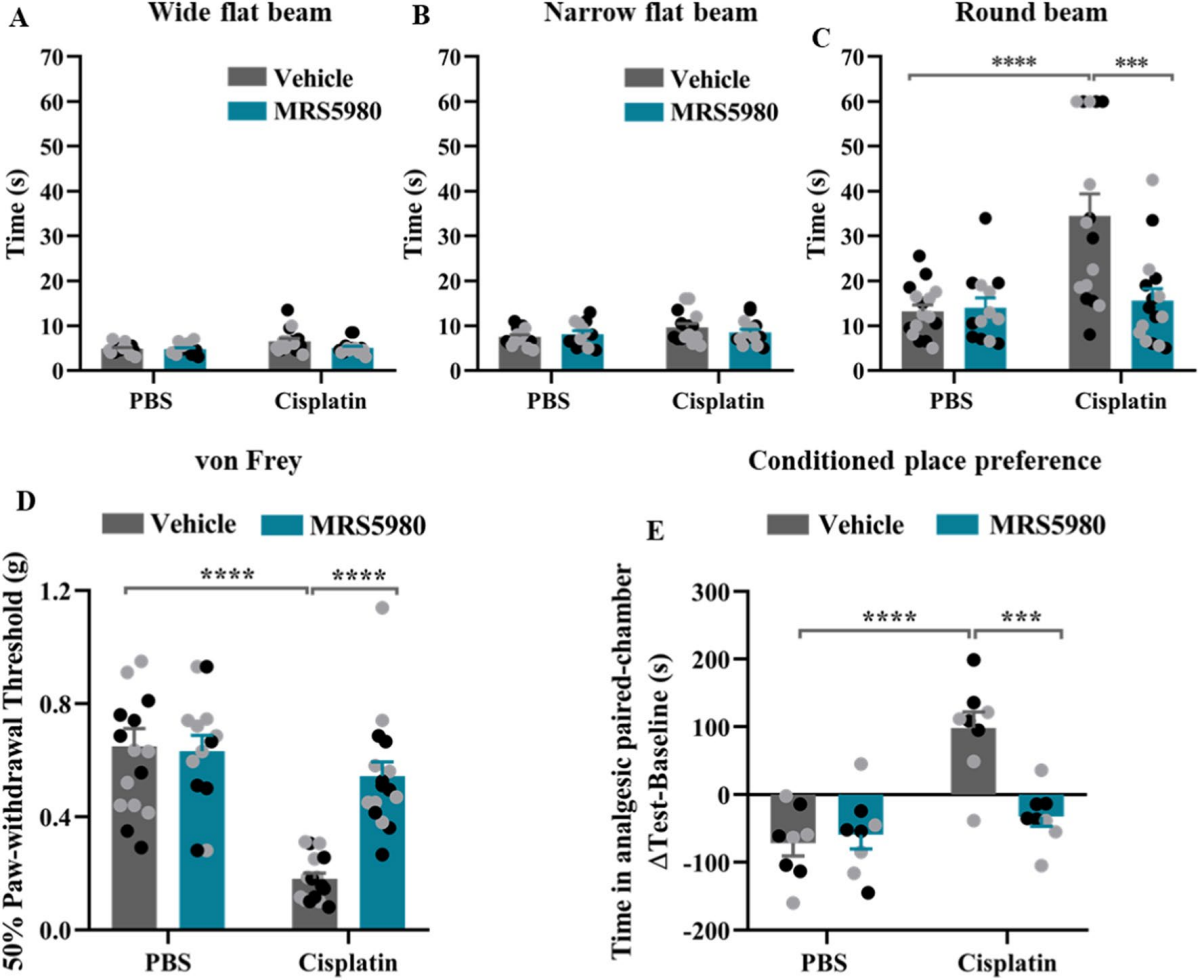
MRS5980 prevents cisplatin-induced sensorimotor deficits, mechanical allodynia and spontaneous pain. Mice were treated with cisplatin and MRS5980 as in Fig. 1A and sensorimotor deficits, mechanical allodynia and spontaneous pain were measured after completion of all cognitive tests. **a–c** Influence of MRS5980 on sensorimotor function in the beam walking test. Male and female mice were tested on three different beams of increasing difficulty. Data are expressed as the time it takes to reach the escape platform at the end of the wide flat (**a**), narrow flat (**b**), and round beam (**c)**. Black circles: male mice, n = 7–8 and grey circles: female mice, n = 6–8 per group. **d** Mechanical sensitivity expressed as 50% paw withdrawal threshold was assessed using von Frey hairs. Black circles: male mice, n = 7–8 and grey circles: female mice, n = 6–8 per group. **e** Effect of MRS5980 on the cisplatin-induced spontaneous pain as measured in the conditional place preference test (CPP). Y-axis indicates the change in time spent in the bright (analgesic-paired chamber) between test and baseline. Black circles: male mice n = 4 and grey circles: female mice, n = 4 per group. Results are expressed as mean ± SEM; Two-way ANOVA with Tukey's post-hoc analysis ***p ≤ 0.001; ****p ≤ 0.0001. No significant sex effects were detected

Patients treated with chemotherapy often experience sensorimotor deficits [19]. To test whether we could model this, we performed a beam walking test. Male and female mice were trained and tested on three different beams with increasing difficulty, wide rectangular, narrow rectangular, and a round beam. We observed no effect of cisplatin on the time taken to cross wide rectangular (Fig. 2A) and narrow rectangular beams (Fig. 2B). However, the cisplatin-treated mice either took more time or were incapable to cross the round beam (Fig. 2C). MRS5980 prevented this sign of cisplatin-induced sensorimotor deficits in both sexes (Fig. 2C).

### The effect of MRS5980 on cisplatin-induced neuropathy

Next, we determined the capacity of MRS5980 to prevent signs of cisplatin-induced peripheral neuropathy in the same group of mice. Cisplatin treatment resulted in mechanical allodynia as assessed using von Frey filaments. MRS5980 prevented cisplatin-induced mechanical allodynia in both sexes (Fig. 2D).

Spontaneous pain and tingling are frequently reported symptoms by patients treated with chemotherapeutics including cisplatin [55]. To investigate the effect of MRS5980 and cisplatin on these symptoms, we used a conditioned place preference (CPP) test with the nerve blocker retigabine as the conditioning stimulus [28]. Cisplatin-treated mice spent more time in the light, retigabine/paired chamber after conditioning (Fig. 2E) indicating spontaneous pain while saline-treated mice had developed a preference for the dark chamber. Mice treated with MRS5980 and cisplatin did not develop a preference for the retigabine-paired light chamber, indicating that these mice did not experience spontaneous pain (Fig. 2E). We did not detect any effect of MRS5980 alone and there were no sex differences (Fig. 2E).

### Cellular localization of A_3_AR expression in the brain

**Fig. 3:**
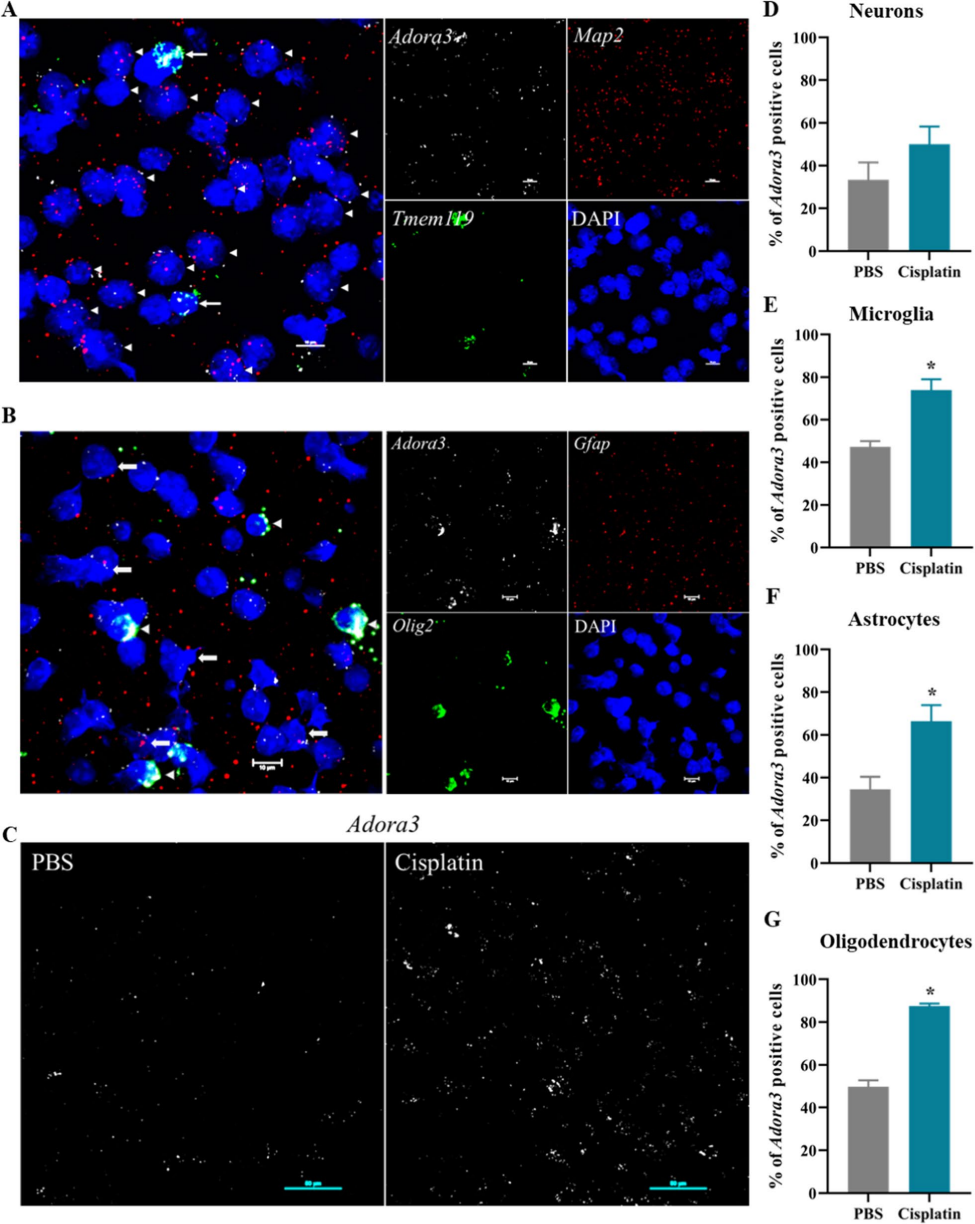
Cellular localization of *Adora3* mRNA expression in the brain after cisplatin treatment. **a**, **b** Expression of *Adora3* mRNA in neurons, microglia, astrocytes, and oligodendrocytes in the cortex of control mice*.* RNAscope in situ hybridization of *Adora3* (white), *Map2* (neuron: red) and *Tmem119* (microglia: green) (**a**); *Adora3* (white), *Gfap* (astrocytes: red) and *Olig2* (Oligodendrocytes: green) (**b**) as analyzed by confocal microscopy; scale bar 10 µm. **c–g** Influence of cisplatin on cellular expression of *Adora3,* 72 h after the first round of cisplatin treatment; scale bar 50 µm. **c** Percentage of *Adora3* positive neurons (**d**), microglia (**e**), astrocytes (**f**), and oligodendrocytes (**g**) in the cortex (n = 3). Data are expressed as mean ± SEM and were analyzed with non-parametric Mann-Witney test; *p < 0.05

The A_3_AR is expressed in the brain, but it is not clear which cells express the receptor. Using RNAscope *in-situ* hybridization analysis of the cortex we detected *Adora3* RNA in neurons, microglia, astrocytes and oligodendrocytes (Fig. 3A, B). Treatment of mice with cisplatin increased *Adora3* expression in microglia, astrocytes and oligodendrocytes (Fig. 3C) with the largest increase in oligodendrocytes (37.8%) followed by astrocytes (31.8%) and microglia (26.6%). The change in *Adora3* mRNA expression in neurons did not reach statistical significance (Fig. 3D–G).

### MRS5980 preserves expression of markers of pre-and post-synaptic integrity in the CA1 region of the hippocampus

**Fig. 4:**
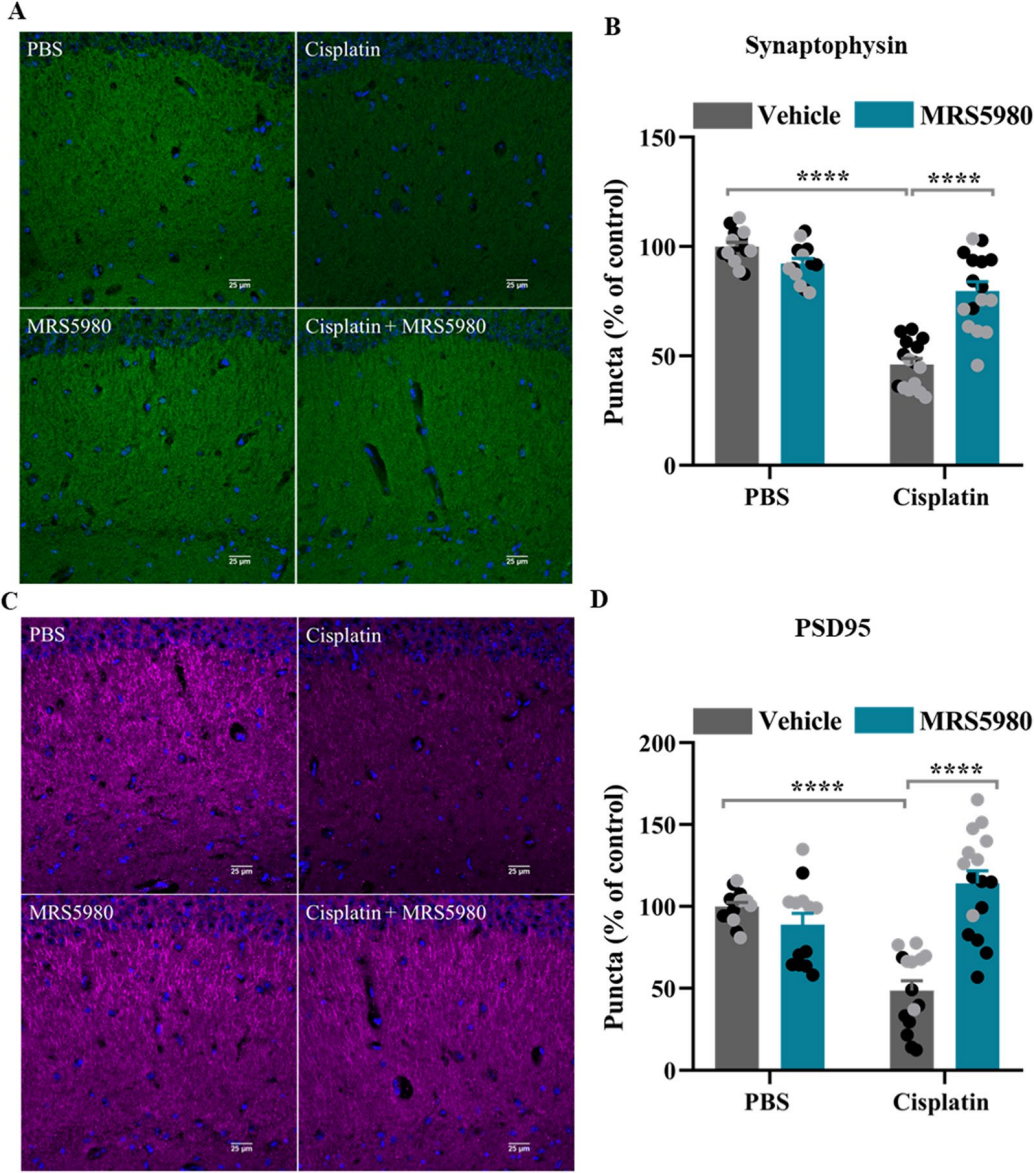
MRS5980 preserves expression of markers of synaptic integrity in cisplatin-treated mice. Male and female mice were treated with cisplatin as in Fig. 2. Eight weeks after the last dose of cisplatin ± MRS9580 brain sections were stained for markers of synaptic integrity. **a** Immunofluorescence images of the pre-synaptic marker synaptophysin in the CA1 region of the hippocampus visualized by confocal microscopy, scale bar 25 µm **b** Quantification of synaptophysin positive puncta expressed as % of control. **c** Immunofluorescence images of the post-synaptic marker PSD95 in the CA1 region visualized by confocal microscopy, scale bar 25 µm. **d** Quantification of the PSD95 positive puncta expressed as % of control. Results are expressed as mean ± SEM; Two-way ANOVA with Tukey's post-hoc analysis ****p ≤ 0.0001. Black circles: male mice n = 7–8 and grey circles: female mice, n = 6–8 per group

Damage to synaptic output is known to contribute to cognitive impairment [29]. In line with a previous study [34], cisplatin decreases the expression of the presynaptic marker synaptophysin (Fig. 4A, B) and the postsynaptic marker PSD95 (Fig. 4C, D) in the CA1 area of the hippocampus. In both sexes MRS5980 prevented the cisplatin-induced loss of synaptophysin (Fig. 4A, B) and PSD95 (Fig. 4C, D) staining in the CA1 region of the hippocampus.

### MRS5980 prevents cisplatin-induced synaptosomal mitochondrial dysfunction and oxidative stress

**Fig. 5:**
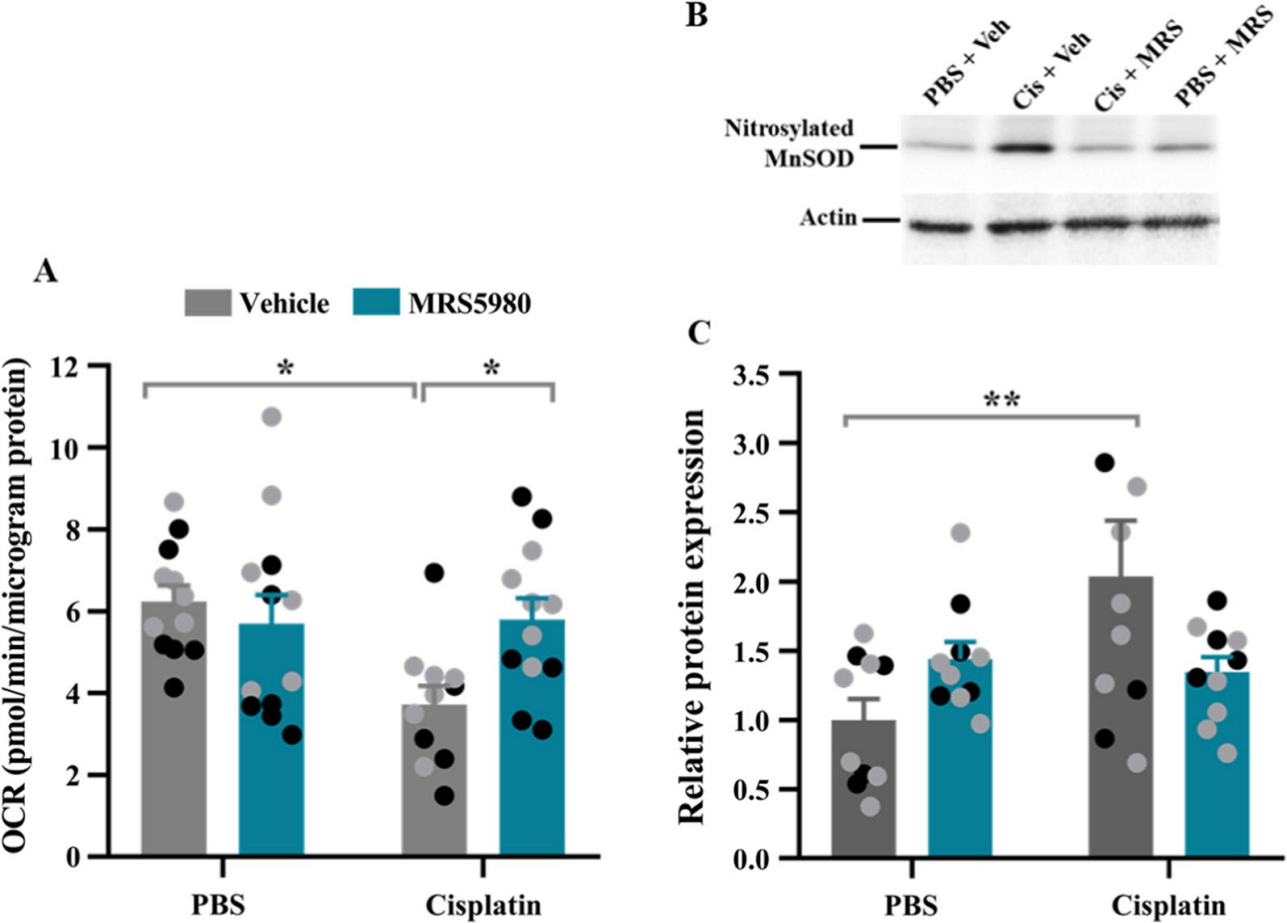
Treatment with MRS5980 prevents cisplatin-induced mitochondrial dysfunction and oxidative stress in the brain. Mice were treated with cisplatin and MRS5980 (0.3 mg/kg) according to Fig. 1A. Brain tissue was collected 4 h after the last dose of cisplatin. **a** Synaptosomal mitochondrial function as assessed by Seahorse analysis. Maximum respiratory capacity (MRC) of mitochondria of brain synaptosomes. Data represent the increase in oxygen consumption rate (OCR) in response to addition of FCCP. Black circles: male mice, n = 5–6 and grey circles: female mice, n = 6 per group. **b**, **c** Western blot analysis of nitrosylated MnSOD in the cortex **(b)** and quantification of the Western blot of nitrosylated MnSOD **(c)**. Black circles: male mice n = 4 and grey circles: female mice, n = 6 per group. Results are expressed as mean ± SEM; Two-way ANOVA with Tukey's post hoc analysis *p ≤ 0.05; **p ≤ 0.01

We previously identified a key role for mitochondrial abnormalities in chemobrain induced by cisplatin [9]. Consistent with these earlier findings, cisplatin treatment reduced the maximum respiratory capacity (MRC) of synaptosomal mitochondria as determined 4 h after the last injection of 2 cycles of cisplatin. As we reported earlier, cisplatin treatment did not change baseline oxygen consumption rate (OCR) normalized for total synaptosomal protein, indicating that mitochondrial content of the synaptosomal fraction was not affected by treatment. Co-administration of MRS5980 decrease in MRC without affecting baseline OCR (Fig. 5A).

To determine whether the cisplatin-induced abnormalities in mitochondrial bioenergetics were associated with signs of oxidative stress, we quantified the level of nitrosylated manganese superoxide dismutase (MnSOD) by immunoprecipitation and western blotting. The level of nitrosylated MnSOD was increased in the cortex of cisplatin-treated mice compared to controls (Fig. 5B, C). MRS5980 prevented the increase in nitrosylated MnSOD in the brains of mice treated with cisplatin (Fig. 5B, C).

### Transcriptome analysis in the cortex after cisplatin and MRS5980

**Fig. 6:**
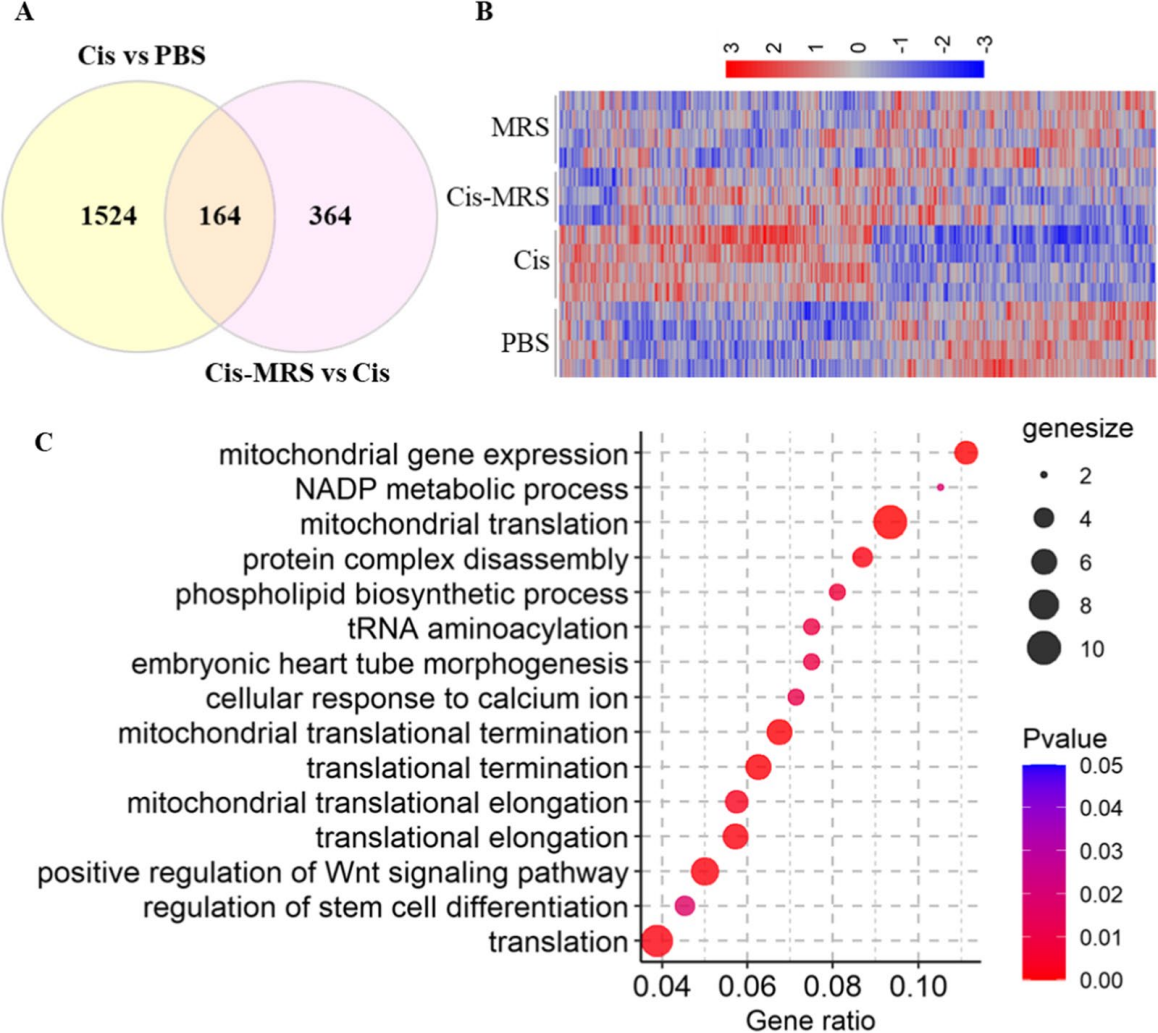
RNAseq analysis of the transcriptome in the cortex after cisplatin and MRS5980. **a** Genes differentially expressed among groups is shown in a Venn diagram. Expression of 1,688 genes was changed in response to cisplatin when compared to the PBS mice (Cis vs PBS; n = 9–12). Expression of 528 genes was changed in response to cisplatin and MRS5980 administration vs. mice treated with cisplatin alone (Cis vs. Cis + MRS5980; n = 9–12). **b** Heatmap showing differential gene expression in response to cisplatin and cisplatin + MRS5980 treatment across all the samples. **c** Gene ontology (GO) biological process enrichment analysis of the 246 genes that were upregulated in the group treated with cisplatin + MRS5980 as compared to cisplatin alone. The size of the dot represents gene count and the color represents the p-value

RNA-seq analysis of cortex samples collected at 4 h after the last dose of cisplatin with or without MRS5980 revealed that cisplatin treatment altered the expression of 1688 genes (P value < 0.05) of which 868 genes were up-regulated and 820 genes were down-regulated by cisplatin (Fig. 6A, B, Additional File 1: Table S1). Co-administration of MRS5980 with cisplatin changed the expression of 528 genes as compared to cisplatin alone (246 up-regulated and 282 down-regulated, P value < 0.05) (Fig. 6A, B, Additional File 2: Table S2). GO biological process enrichment analysis of the 246 genes that were upregulated in the group treated with cisplatin + MRS5980 as compared to cisplatin alone identified mainly pathways involved in regulation of translation and development (Wnt signaling and stem cell differentiation), and in phospholipid biosynthesis (Fig. 6C).

**Fig. 7:**
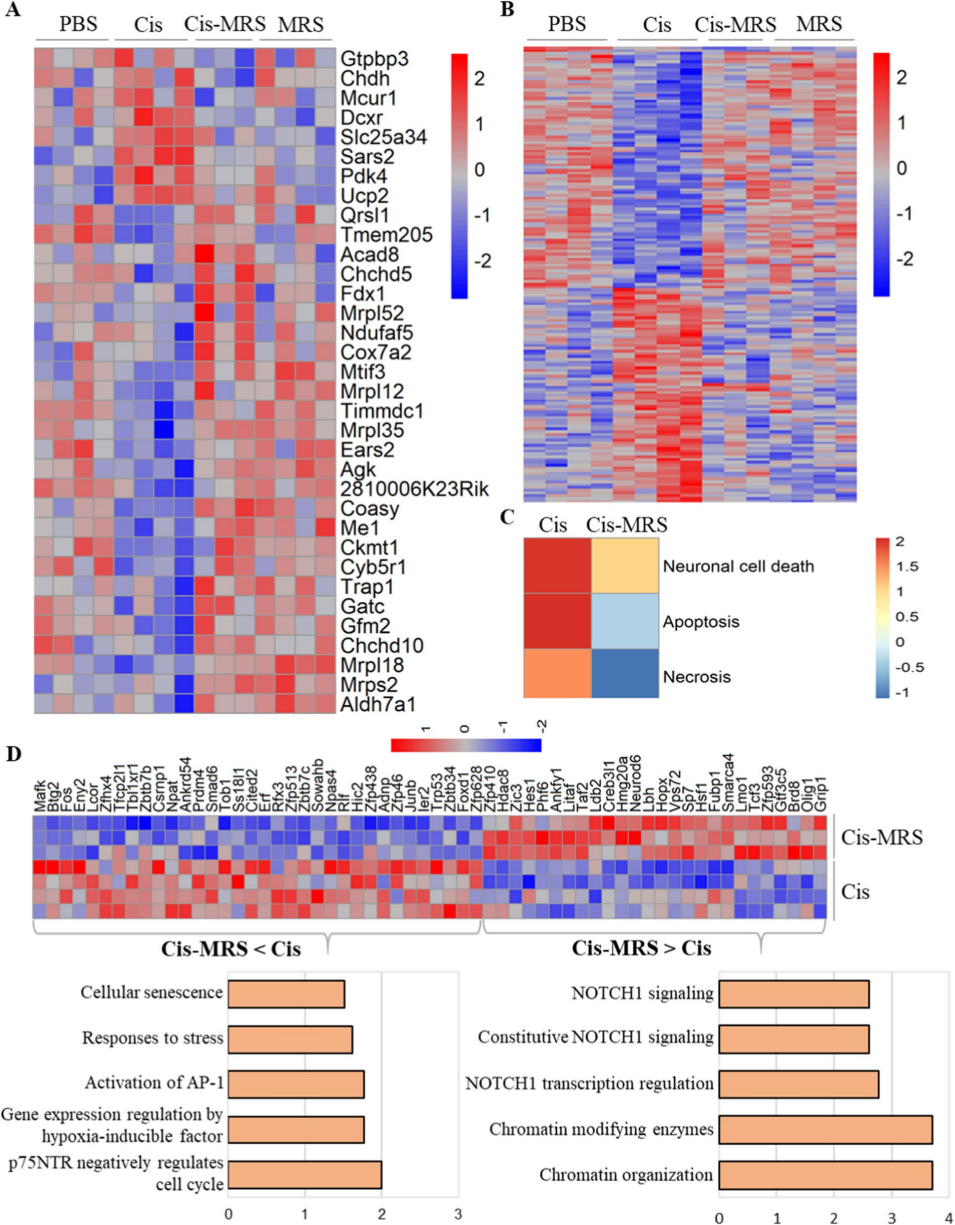
MRS5980 prevents cisplatin-induced changes in mitochondria related genes, neuronal cell death, apoptosis and necrosis. **a** Heatmap showing the expression pattern of mitochondrial related genes across four treatment groups. **b** Heatmap of the 164 overlapping genes between cisplatin vs PBS and cisplatin + MRS5980 vs cisplatin comparisons. **c** Top 3 functional enrichment pathways from cisplatin vs PBS and cisplatin + MRS5980 vs cisplatin comparisons. **d** The heatmap shows the expression pattern of transcription regulators from cisplatin vs cisplatin + MRS5980 comparison. The bar plot shows the top GO biological process identified based on transcription regulators

In view of the known role of mitochondrial bioenergetic deficits in cognitive impairments induced by cisplatin [8, 9], we assessed the expression of mitochondria-related genes. The results in Fig. 7A show that cisplatin altered expression of 34 mitochondria-related genes. Interestingly, these changes were mostly prevented by co-administration of MRS5980. Many of the genes of which the cisplatin-induced changes were prevented by co-administration of MRS5980 were also altered in response to administration of MRS5980 alone to control mice (Fig. 7A).

Although MRS5980 fully prevented cisplatin-induced cognitive deficits and structural damage to the brain, MRS5980 administration prevented the cisplatin-induced change in only 164 genes out of the 1688 that were altered by cisplatin alone (Overlap in Fig. 6A; Heatmap Fig. 7B). Thus, expression of 1524 genes were significantly affected by cisplatin and not prevented by MRS5980 co-administration. Functional enrichment analysis of the genes that increased by cisplatin alone and not affected by MRS5980 identified Neuronal cell death, Apoptosis and Necrosis as the pathways with the highest increase (Z scores > 1.5 (Fig. 7C).

Moreover, comparison of the expression of transcription regulator genes between the cisplatin and cisplatin + MRS5980 treated groups showed that the genes that are decreased by administration of cisplatin + MRS5980 are enriched for pathways related to cellular stress, senescence and hypoxia in the top five (Fig. 7D, left). In contrast, the genes that are increased by MRS5980 administration together with cisplatin are enriched in genes related to NOTCH1 signaling and chromatin modification/ organization (Fig. 7D, right). These findings indicate that the beneficial effect of MRS5980 is mediated in part via activation of repair pathways.

### Reversal of cisplatin-induced behavioral deficits by MRS5980

**Fig. 8:**
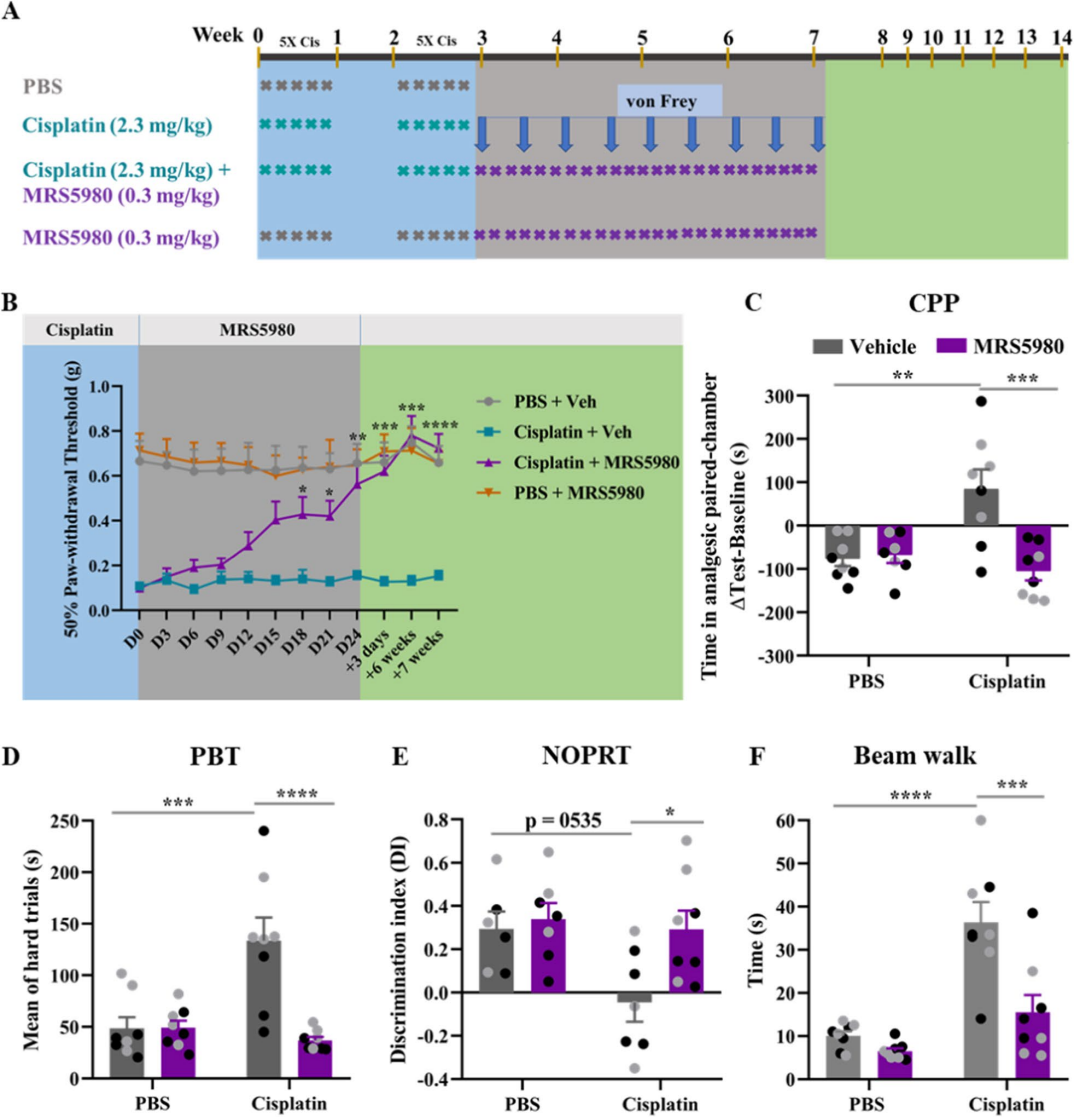
MRS5980 reverses cisplatin-induced behavioral deficits. **a** Male (n = 4/group) and female (n = 4/group) mice were treated with 2 cycles of cisplatin (2.3 mg/kg/day) followed by 24 doses of MRS5980 (0.3 mg/kg/day) starting one day after the last dose of cisplatin. **b** Mechanical allodynia was measured twice a week using von Frey hairs. Results are expressed as mean ± SEM; Two-way ANOVA (repeated measures) with Tukey's post hoc analysis *p ≤ 0.05; **p ≤ 0.01; ***p ≤ 0.0)1; ****p ≤ 0.0001. **c** Effect of MRS5980 on cisplatin-induced spontaneous pain as measured in CPP test after 4 weeks after the last dose of MRS5980. Y-axis indicates the change in time spent in the bright (analgesic-paired chamber) between baseline and test. **d** Effect of MRS5980 after completion of cisplatin treatment on executive functioning measured using the PBT as assessed one week after the last dose of MRS5980. Mean of the time in seconds it takes to enter the dark compartment in the hard trial of the PBT. **e** Effect of MRS5980 after completion of cisplatin treatment on spatial and working memory. Preference for the novel object in the NOPRT is presented as discrimination index (DI) as in Fig. 1C. **f** Influence of MRS5980 on cisplatin-induced sensorimotor deficits. Mice were tested on three different beams of increasing difficulty and the time to cross the round beam is depicted. Results are expressed as mean ± SEM; Two-way ANOVA with Tukey's post hoc analysis *p ≤ 0.05; **p ≤ 0.01; ***p ≤ 0.001; ****p ≤ 0.0001. Black circles (male mice) and grey circles (female mice)

RNAseq analysis revealed that MRS5980 did not only prevent cisplatin-induced changes in gene expression but also may activate reparative pathways. Therefore, we tested whether MRS5980 could *revers*e the neurotoxic side effects of cisplatin. Male and female mice were treated with cisplatin followed by 24 daily injections of MRS5980 (0.3 mg/kg/day, i.p.) starting one day after the last dose of cisplatin (Fig. 8A). We monitored mechanical allodynia over time. There was no immediate effect of MRS5980 on cisplatin-induced mechanical allodynia. However, after twelve doses, the first signs of a reduction in mechanical allodynia were detected, and after 18 doses statistical significance was reached. We continued dosing until day 24 when full reversal of mechanical allodynia was observed (Fig. 8B). Notably, reversal of mechanical allodynia was maintained until at least 7 weeks after the last dose of MRS5980, indicating resolution of allodynia rather than analgesic effects of MRS5980 (Fig. 8B). In support of this conclusion, MRS5980-treated mice did not show signs of spontaneous pain in the CPP test 4 weeks after the completion of MRS5980 treatment. The data in Fig. 8C shows that the 24 daily doses of MRS5980 treatment had fully reversed cisplatin-induced spontaneous pain in both sexes (Fig. 8C).

To determine whether 24 daily doses of MRS5980 also reversed chemobrain, we performed the PBT and NOPRT starting one week after the completion of MRS5980 treatment. At this time point, 5 weeks after the last dose of cisplatin, cisplatin-treated mice still showed a deficit in both tests. Administration of 24 daily doses of MRS5980 days after completion of cisplatin treatment completely reversed the deficit in executive functioning and in spatial and working memory in male and female mice (Fig. 8D, E). Assessment of sensorimotor function in the beam walking test at 7 weeks after the last dose of cisplatin revealed that cisplatin-treated mice were still impaired. Notably, 24 doses of MRS5980 normalized performance in the beam walking test (Fig. 8F). Treatment with MRS5980 alone in the absence of cisplatin did not affect behavior in any of the tests (Fig. 8B–F). Taken together, these findings show that MRS5980, not only prevents but also reverses cisplatin-induced cognitive impairments, sensorimotor deficits, mechanical allodynia, and spontaneous pain.

## Discussion

With increasing numbers of cancer survivors, there has been growing interest in managing the long-term side effects of chemotherapy, especially cognitive impairment, sensorimotor deficits, and neuropathic pain. In pursuit of interventions to prevent and/or reverse these adverse side effects of cancer treatment, it is imperative that the intervention does not interfere with the efficacy of cancer treatment. Preclinical and clinical studies indicate that A_3_AR agonists meet this requirement; A_3_AR expression is increased in multiple tumors and A_3_AR agonists inhibit the growth of cancer cells in vitro and reduce tumor growth in vivo [15]. Clinically, the A_3_AR agonist Cl-IB-MECA (Namodenoson) increased survival in patients with advanced hepatocellular carcinoma without serious adverse effects [20, 45]. Previous work has demonstrated that A_3_AR agonists protect against brain injury and cognitive deficits in models of cerebral ischemia, seizure, and traumatic brain injury [14, 51, 52]. Accumulating evidence indicates a critical role of A_3_AR in pain regulation and agonists are emerging as promising candidates for the treatment of inflammatory pain and neuropathic pain [5, 10, 23, 24, 31, 50]. We tested the therapeutic potential of a recently developed highly selective (10,000-fold selectivity over other subtypes of adenosine receptors) A_3_AR agonist, MRS5980 [21], in our well-established mouse model of cisplatin-induced cognitive deficits and peripheral neuropathy [7, 8, 26, 36]. To the best of our knowledge, this study is the first to describe that MRS5980 not only prevents but also reverses cisplatin-induced cognitive impairments, sensorimotor deficits, and peripheral neuropathic pain. At the structural level in the brain, we show that co-administration of MRS5980 with cisplatin prevents the loss of the markers of synaptic integrity synaptophysin and PSD95. Mechanistically, co-administration of MRS5980 prevented cisplatin-induced synaptic mitochondrial dysfunction, and oxidative stress. RNAseq analysis showed that cisplatin-induced changes in mitochondrial function related genes were prevented by co-administration of MRS5980. Moreover, MRS5980 activated NOTCH1 signaling and chromatin remodeling related pathways in the brain indicating that activation of repair pathways likely contributes to its protective effect. Finally, we show that administration of 24 daily doses of MRS5980 after completion of cisplatin treatment resulted in resolution of signs of cognitive impairments, sensorimotor deficits and neuropathic pain and this beneficial effect was maintained for at least 7 weeks after the last dose of MRS5890. Collectively, our findings demonstrate that the A_3_AR agonist MRS5890 protects against and reverses cisplatin-induced cognitive impairments, sensorimotor deficits and neuropathic pain. Clinical translation of these findings would benefit a growing number of cancer survivors suffering from these long-lasting neurotoxic side effects of chemotherapy.

Previous work from our group reported that the neurotoxic side effects of cisplatin are caused by structural and functional damage to mitochondria in brain synaptosomes and dorsal root ganglion neurons [9, 38]. Cisplatin rapidly induces mitochondrial accumulation of p53 in brain and dorsal root ganglia. Co-administration of PFT-µ, an inhibitor of mitochondrial p53 accumulation prevents cisplatin-induced cognitive impairment and peripheral neuropathy [9, 38]. Here we show that the A_3_AR agonist prevents cisplatin-induced changes in expression of genes related to mitochondrial function and protects against brain synaptosomal mitochondrial dysfunction and the associated oxidative stress as evidenced by prevention of the cisplatin-induced increase in nitrosylated MnSOD.

Previous studies indicated A_3_AR agonists employ a central mode of action to suppress neuropathic pain [31] and reduce ischemic and traumatic brain injury [14, 52]. A_3_AR is present in the brain, but single cell RNA sequencing or RT PCR analysis on laser-dissected neurons led to conflicting data regarding the presence of A_3_AR mRNA in neurons [32, 46]. Using cell-specific probes and RNA in situ hybridization, our results clearly demonstrate abundant expression of A_3_AR RNA in neurons, microglia, astrocytes, and oligodendrocytes and upregulation in response to cisplatin. The increased A_3_AR expression in cisplatin-treated brains may well contribute to the protective effect of the A_3_AR agonist.

A_3_AR agonists have been shown to induce anti-inflammatory effects that are thought to be mediated via suppression of NF-kB activity [20]. The protective effects of A_3_AR agonists in models of ischemic or traumatic brain injury and neuropathic pain have been attributed to suppression of neuroinflammation [5, 10, 14, 52]. However, our current and previous transcriptomic analysis using RT-PCR of select genes or unbiased RNA seq approaches did not identify signs of inflammation in the brain of mice treated with cisplatin as determined by RNA seq analysis or qRT-PCR during and immediately after cisplatin treatment, or after completion of behavioral analysis [1, 7, 9]. Moreover, we did not detect morphological changes in astrocytes or microglia during and after cisplatin treatment [6, 57]. Therefore, it is unlikely that the beneficial effects of the A_3_AR agonist in our model of cisplatin-induced cognitive deficits are mediated primarily via suppression of neuroinflammation.

The anti-tumor effects of A_3_AR activation involves multiple signaling pathways including NF-κB and Wnt signaling pathways leading to inhibition of cell growth regulatory genes, such as c-myc and cyclin D1, and apoptosis of tumor cells [16, 17]. Functional enrichment analysis of the genes that were differentially expressed in samples from mice treated with cisplatin + MRS5980 vs cisplatin alone identified “positive regulation of Wnt signaling pathways” and pathways related to protein synthesis. Wnt signaling has been shown to play an important role in repair especially in the survival of neural stem cells/progenitor cells and regulation of BDNF release, a neurogenic niche which is mandatory for intact cognitive capabilities [25, 41]. We and others showed that cisplatin treatment depletes the neurogenic niche [9, 42], therefore upregulation of Wnt signaling in response to MRS5980 could be an instrumental signaling pathway to restore cognition. MRS5980 activates pathways related to NOTCH1 signaling and chromatin modification and organization, which again are positively involved in wound healing processes and repair [44, 48]. Analysis of the upstream regulators shows that MRS5980 treatment suppresses pathways related to cellular senescence, oxidative stress and activation of AP-1, all of which are involved in wound healing processes and the maintenance and repair of homeostasis [11, 54]. Collectively, these findings indicate that MRS5980 does not only prevent cisplatin-induced cognitive deficits by simply preventing the damaging effects, but also stimulates pathways that may well initiate and/or maintain repair mechanisms including the repair of mitochondrial function. Notably, administration of MRS5980 to control mice induced and suppressed many of the same genes that were also regulated by giving MRS5980 to cisplatin-treated mice.

The finding in the RNAseq analysis that MRS5980 may induce restorative pathways in mice with cisplatin-induced cognitive decline and neuropathy led us to investigate the effect of MRS5980 treatment *after completion* of chemotherapy. Earlier studies have shown that the A_3_AR agonist IB-MECA rapidly suppresses hyperalgesia in mice with neuropathic pain induced by chronic constriction injury to the sciatic nerve [5]. This rapid analgesic effect was mediated via the release of IL10 by T cells [13]. Moreover, prevention of chemotherapy-induced allodynia by co-administration of an A_3_AR agonist is IL10 mediated as well [53]. Notably, we showed recently that the spontaneous resolution of cisplatin-induced neuropathy is dependent on IL10 signaling as well [27]. Moreover, the resolution of cisplatin-induced neuropathic pain in response to nasal administration of mesenchymal stem cells was also mediated by IL10 [3], indicating that IL10 signaling to peripheral sensory neurons could represent a common mechanism underlying resolution of neuropathic pain. It remains to be determined whether IL10 signaling is involved in the slowly developing but persistent reversal of established cisplatin-induced neuropathy.

## Conclusions

Here we show that the A_3_AR agonist, MRS5980, prevents cisplatin-induced cognitive impairments, sensorimotor deficits, and neuropathic pain. In the brain, MRS5980 preserved pre-and post-synaptic proteins in the CA1 subregion of the hippocampus. The beneficial effects of MRS5980 are associated with the prevention of cisplatin-induced mitochondrial dysfunction in synaptosomes and preservation of synaptic integrity. Its primary beneficial effect may be through prevention of cisplatin-induced mitochondrial dysfunction. In addition, based on our RNA sequencing analysis we propose that MRS5980 may add to the restoration of brain function by exerting a regenerative effect, irrespective of cisplatin treatment. A_3_AR agonists are already used clinically to treat inflammatory disorders and as an add-on therapy for cancer. Therefore, clinical translation of our finding that a highly selective A_3_AR agonist can prevent and reverse chemotherapy-induced cognitive decline, sensorimotor deficits and peripheral neuropathy should be feasible.

## Supplementary Information

**Additional file1: Table S1. **Differentially expressed genes (PBS vs Cisplatin).

**Additional file 2: Table S2. **Differentially expressed genes (Cisplatin vs Cisplatin + MRS5980).

**Additional file 3: Supplementary figure S1. **Dose finding experiment for the effect of the A3AR agonist MRS5980 on chemobrain. **Supplementary figure S2**: No effect of treatments on performance in easy and intermediate PBT trials, total interaction time in the NOPRT, and total arm entries in the Y-maze.

## Data Availability

The datasets used and/or analyzed during the current study are available from the corresponding author on reasonable request.

## Abbreviations

A_3_AR: A_3_ adenosine receptor
CA1: Cornu ammonis 1
CPP: Conditioned place preference
CICI: Chemotherapy-induced cognitive impairments
CNS: Central nervous system
DMSO: Dimethyl sulfoxide
DRG: Dorsal root ganglia
FDA: Food and drug administration
GFAP: Glial fibrillary acidic protein
IL10: Interleukin 10, NK cell: natural killer cell
IENF: Intra-epidermal nerve fibers
IP: Intraperitoneally
NOPRT: Novel object place recognition test
OCR: Oxygen consumption rate
PBS: Phosphate buffered saline
PBT: Puzzle box test
PSD95: Postsynaptic density protein 95
RNA: Ribonucleic acid
ROI: Regions of interest
TEM: Transmission electron microscope
