# Supplementary material for: Targeting the A3 adenosine receptor to prevent and reverse chemotherapy-induced neurotoxicities in mice
Source: Acta Neuropathol Commun. 2022 Jan 29;10:11. doi: 10.1186/s40478-022-01315-w (PMC8800287; doi:10.1186/s40478-022-01315-w)
Supplement: Supplementary file 3 — Additional file 3: Supplementary figure S1. Dose finding experiment for the effect of the A3AR agonist MRS5980 on chemobrain. Supplementary figure S2: No effect of treatments on performance in easy and intermediate PBT trials, total interaction time in the NOPRT, and total arm entries in the Y-maze. [file 40478_2022_1315_MOESM3_ESM.docx]

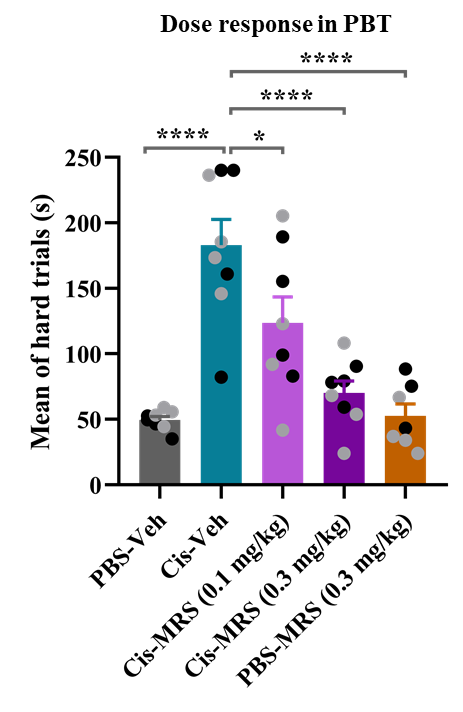


**Supplementary figure S1: Dose finding experiment for the effect of the A3AR agonist MRS5980 on chemobrain.** Dose response in puzzle box test (PBT). Effect of two doses of MRS5980 (0.1 and 0.3 mg/kg) on cisplatin-induced deficits in executive function as assessed using the puzzle box test (PBT) (black circles: male mice, n= 4 and grey circles: female mice, n= 4 per group). The results are expressed as mean± SEM and were analyzed with one-way ANOVA followed by Tukey's multiple comparisons test; **p* < 0.05, *****p* < 0.0001.


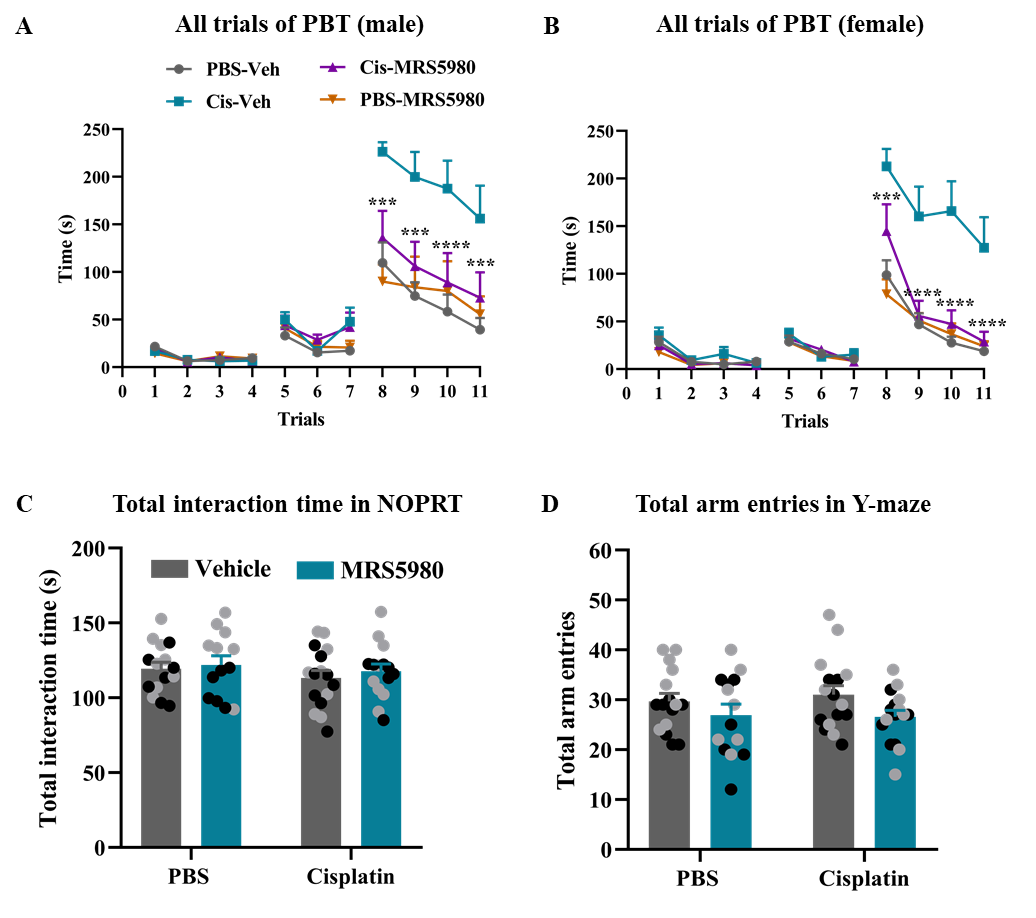


**Supplementary figure S2: No effect of treatments on performance in easy and intermediate PBT trials, total interaction time in the NOPRT, and total arm entries in the Y-maze.**

Effect of MRS5980 in cisplatin-treated **(A)** male mice and **(B)** female mice in all trials of the PBT. Results are expressed as mean ± SEM. **(C)** Total interaction time in NOPRT expressed as time spent with the novel plus time spent with the familiar object during test. **(D)** Total number of arm entries in Y-maze. Results are expressed as mean ± SEM. Black circles (male mice) and grey circles (female mice).
